# Reply: *KRAS* status analysis and anti-EGFR therapies: is comprehensiveness a biologist's fancy or a clinical necessity?

**DOI:** 10.1038/sj.bjc.6605583

**Published:** 2010-02-16

**Authors:** C Cremolini, A Ruzzo, F Graziano, A Falcone, F Loupakis

**Affiliations:** 1 UO Oncologia Medica 2 Universitaria, Azienda Ospedaliera-Universitaria Pisana, Istituto Toscano Tumori, Via Roma, 67, Pisa 56100, Italy; 2 Dipartimento di Scienze Biomolecolari, Università di Urbino, Urbino, Italy; 3 UO Oncologia Medica, Ospedale di Pesaro, Pesaro, Italy; 4 Dipartimento di Oncologia, dei Trapianti e delle Nuove Tecnologie in Medicina, Università di Pisa, Pisa, Italy

**Sir,**

We thank Dr Lopez-Crapez *et al* 2010 to have taken cue from our publication (Loupakis *et al*, 2009) to highlight a really living matter: what does ‘*KRAS*-mutated tumor’ mean? Both methodological and technical issues contribute to make the answer extremely complex. Two crucial aspects deserve consideration: (i) many *KRAS* mutations that occur with low frequencies have not been included in the *post*-*hoc* analyses of large phase III randomised trials; (ii) mutation-enriched techniques increase the percentage of *KRAS*-mutated specimens by about 15%, when compared with direct sequencing (Marchetti and Gasparetti, 2009).

Recent studies have shown that retrospective experiences are indispensable starting points to identify promising molecular tools, that need to be further evaluated in adequate trials and to be, if appropriate, introduced in clinical practice. To this end, the example of *BRAF* mutations, rapidly translated from retrospective series (Di Nicolantonio *et al*, 2008) to clinical guidelines (http://www.nccn.org) despite the inconclusive results of the *post-hoc* analysis of CRYSTAL trial (Rougier *et al*, 2009), is emblematic. It is, therefore, undeniable that the strength of retrospectively acquired evidences draws from their reproducibility. Recently presented data from the broad experience of the European Consortium, which included 723 retrospectively analysed specimens (Tejpar and De Roock, 2009), actually strengthen our results about the negative predictive role of *KRAS* codon 61 mutations.

On the other hand, more ambiguous findings have been reported with regard to *KRAS* A146T mutation, although its activating power was strongly suggested by *in vitro* mutagenesis assays (Feig and Cooper, 1988) and confirmed by mutational screening analyses, performed on wide series of colorectal cancers (Edkins *et al*, 2006). According to data from the European Consortium, such somatic mutation, found in 13 analysed samples, was not mutually exclusive with other *KRAS* activating alterations and was not clearly linked with lack of response to cetuximab plus irinotecan, thus raising perplexity about its negative predictive impact.

Hence, in conclusion, is the comprehensive detection of all potential *KRAS* alterations, including those ‘not described before in any cancer type’, a *biologist's fancy* or a *clinical necessity*? Probably neither of them. Nowadays, in daily practice, the definition of ‘*KRAS*-mutated tumor’ leads physicians to deny to metastatic colorectal cancer patients a class of efficacious drugs. Therefore, the relevance of rare or complex *KRAS* variants, of which the activating properties and actual predictive power can be hypothesised, but not clearly stated, should be evaluated with caution.

The comprehensive analysis of *KRAS* gene represents, instead, an *inescapable investigational need*, with the aim to better characterise the biology of colorectal cancer and to opportunely verify the predictive potential of *KRAS* rare variants.
